# 4-[4-(Diethyl­amino)benzyl­ideneamino]-4*H*-1,2,4-triazole

**DOI:** 10.1107/S1600536808009100

**Published:** 2008-05-03

**Authors:** Jian Xin Pan, Ju Zhou Zhang, Qian Wang Chen

**Affiliations:** a Hefei National Laboratory for Physical Sciences at Microscale, and Department of Materials Science & Engineering, University of Science and Technology of China, Hefei 230026, People’s Republic of China

## Abstract

The title compound, C_13_H_17_N_5_, is a Schiff base synthesized by the reaction of 4-amino-4*H*-1,2,4-triazole and 4-(diethyl­amino)benzaldehyde. The triazole ring forms a dihedral angle of 5.77 (16)° with the benzene ring. The crystal structure is stabilized by an inter­molecular C—H⋯N hydrogen bond.

## Related literature

For related literature, see: Zhu *et al.* (2000 ▶), Atalay *et al.* (2003 ▶); Petek *et al.* (2004 ▶); Brasselet *et al.* (1999 ▶); Cornelissen *et al.* (1992 ▶); Demirbs & Ugurluoglu Demirbas (2002 ▶); Fujigaya *et al.* (2003 ▶); Garcia *et al.* (1997 ▶); Kahn & Martinez (1998 ▶); Moliner *et al.* (2001 ▶); Tozkoparan *et al.* (2000 ▶); Turan-Zitouni *et al.* (1999 ▶).
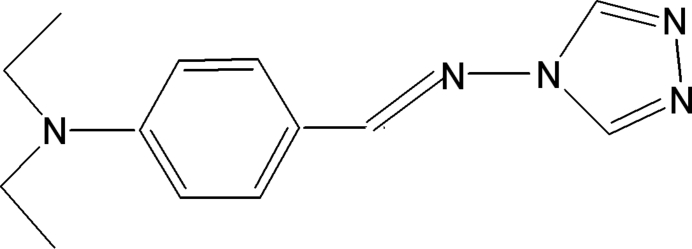


## Experimental

### 

#### Crystal data

C_13_H_17_N_5_
*M* *_r_* = 243.32
Orthorhombic, 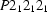

*a* = 7.740 (3) Å
*b* = 9.238 (4) Å
*c* = 18.497 (7) Å
*V* = 1322.5 (9) Å^3^
*Z* = 4
Mo *K*α radiation
μ = 0.08 mm^−1^
*T* = 293 (2) K
0.37 × 0.35 × 0.11 mm

#### Data collection

Bruker APEX2 CCD area-detector diffractometer
Absorption correction: multi-scan (*SADABS*; Sheldrick, 1996 ▶) *T*_min_ = 0.972, *T*_max_ = 0.992
6650 measured reflections
1359 independent reflections
895 reflections with *I* > 2σ(*I*)
*R*_int_ = 0.075

#### Refinement

*R*[*F* ^2^ > 2σ(*F* ^2^)] = 0.059
*wR*(*F* ^2^) = 0.131
*S* = 1.09
1359 reflections
165 parameters
H-atom parameters constrained
Δρ_max_ = 0.19 e Å^−3^
Δρ_min_ = −0.17 e Å^−3^

### 

Data collection: *APEX2* (Bruker, 2005 ▶); cell refinement: *APEX2*; data reduction: *APEX2*; program(s) used to solve structure: *SIR97* (Altomare *et al*., 1999 ▶); program(s) used to refine structure: *SHELXL97* (Sheldrick, 2008 ▶); molecular graphics: *SHELXTL* (Sheldrick, 2008 ▶); software used to prepare material for publication: *WinGX* (Farrugia, 1999 ▶).

## Supplementary Material

Crystal structure: contains datablocks I, global. DOI: 10.1107/S1600536808009100/rz2200sup1.cif

Structure factors: contains datablocks I. DOI: 10.1107/S1600536808009100/rz2200Isup2.hkl

Additional supplementary materials:  crystallographic information; 3D view; checkCIF report

## Figures and Tables

**Table 1:** Hydrogen-bond geometry (Å, °)

*D*—H⋯*A*	*D*—H	H⋯*A*	*D*⋯*A*	*D*—H⋯*A*
C1—H1⋯N1^i^	0.93	2.43	3.296 (7)	155

Symmetry code: (i) 
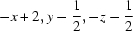
.

**Fig. 1.**
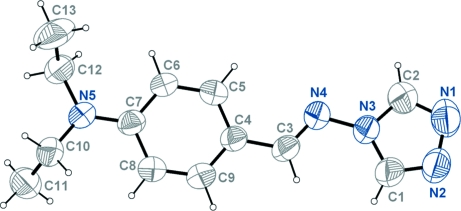
The molecular structure of the title compound, with the atom-numbering scheme. Displacement ellipsoids are drawn at the 50% probability level.

## supplementary crystallographic information

### Comment

Recent interest in substituted 1,2,4-triazoles has arisen in part from their transition metal complexes with intriguing structures and specific magnetic properties (Garcia *et al.*, 1997; Kahn & Martinez, 1998; Moliner *et al.*, 2001; Fujigaya *et al.*, 2003). In addition, many compounds containing a 1,2,4-triazole unit display a broad range of biological and pharmacological activities, finding application as anti-inflammatory (Tozkoparan *et al.*, 2000), antitumour (Demirbs & Ugurluoglu Demirbas, 2002), analgesic (Turan-Zitouni *et al.*, 1999), antibacterial and antiviral agents (Cornelissen *et al.*, 1992). In a continuation of our interest in the chemical and pharmacological properties of triazole derivatives, we have synthesized the title compound and report here its crystal structure.

The molecular structure and the atom-numbering scheme of the title compound are shown in Fig. 1. In the molecule, all bond lengths and angles are within normal ranges and comparable with the reported values (Atalay *et al.*, 2003; Zhu *et al.*, 2000). In the triazole ring, the N2?C1 and N1?C2 bonds display double-bond character, with bond distances of 1.288 (6) and 1.313 (6) Å, respectively. The 1,2,4-triazole ring is strictly planar (maximum displacement 0.006 (5) Å for C2) and forms a dihedral angle of 5.77 (16) °. The crystal packing is stabilized by an intermolecular C—H···N hydrogen bonding interaction (Table 1).

### Experimental

A mixture of 4-amino-l,2,4-triazole (0.88 g, 10 mmol) and 4-(diethylamino)benzaldehyde (1.77 g, 10 mmol), which was prepared by standard procedures (Brasselet *et al.*, 1999), was dissolved in ethanol (180 ml) and stirred for 1 h. Single crystals suitable for X-ray diffraction analysis were obtained by slow evaporation of the ethanol solution.

### Refinement

The H atoms were positioned geometrically, with C—H = 0.93, 0.97 and 0.96 Å for aromatic, methylene and methyl H atoms, respectively, and constrained to ride on their parent atoms, with *U*_iso_(H) = *xU*_eq_(C), where *x* = 1.5 for methyl H, and *x* = 1.2 for all other H atoms. In the absence of significant anomalous scattering effects, Friedel pairs were merged.

### Crystal data

**Table:**

C_13_H_17_N_5_	*F*_000_ = 520
*M**_r_* = 243.32	*D*_x_ = 1.222 Mg m^−^^3^
Orthorhombic, *P*2_1_2_1_2_1_	Mo *K*α radiation λ = 0.71073 Å
Hall symbol: P 2ac 2ab	Cell parameters from 870 reflections
*a* = 7.740 (3) Å	θ = 2.5–20.5º
*b* = 9.238 (4) Å	µ = 0.08 mm^−^^1^
*c* = 18.497 (7) Å	*T* = 293 (2) K
*V* = 1322.5 (9) Å^3^	Block, yellow
*Z* = 4	0.37 × 0.35 × 0.11 mm

### Data collection

**Table:**

Bruker APEX2 CCD area-detector diffractometer	1359 independent reflections
Radiation source: fine-focus sealed tube	895 reflections with *I* > 2σ(*I*)
Monochromator: graphite	*R*_int_ = 0.075
*T* = 293(2) K	θ_max_ = 25.0º
φ and ω scans	θ_min_ = 2.5º
Absorption correction: multi-scan(SADABS; Sheldrick, 1996)	*h* = −9→9
*T*_min_ = 0.972, *T*_max_ = 0.992	*k* = −10→10
6650 measured reflections	*l* = −21→13

### Refinement

**Table:**

Refinement on *F*^2^	Secondary atom site location: difference Fourier map
Least-squares matrix: full	Hydrogen site location: inferred from neighbouring sites
*R*[*F*^2^ > 2σ(*F*^2^)] = 0.060	H-atom parameters constrained
*wR*(*F*^2^) = 0.131	*w* = 1/[σ^2^(*F*_o_^2^) + (0.0187*P*)^2^ + 0.429*P*] where *P* = (*F*_o_^2^ + 2*F*_c_^2^)/3
*S* = 1.09	(Δ/σ)_max_ < 0.001
1359 reflections	Δρ_max_ = 0.19 e Å^−^^3^
165 parameters	Δρ_min_ = −0.17 e Å^−^^3^
Primary atom site location: structure-invariant direct methods	Extinction correction: none

### Special details

**Table:**

Geometry. All e.s.d.'s (except the e.s.d. in the dihedral angle between two l.s. planes) are estimated using the full covariance matrix. The cell e.s.d.'s are taken into account individually in the estimation of e.s.d.'s in distances, angles and torsion angles; correlations between e.s.d.'s in cell parameters are only used when they are defined by crystal symmetry. An approximate (isotropic) treatment of cell e.s.d.'s is used for estimating e.s.d.'s involving l.s. planes.
Refinement. Refinement of *F*^2^ against ALL reflections. The weighted *R*-factor *wR* and goodness of fit *S* are based on *F*^2^, conventional *R*-factors *R* are based on *F*, with *F* set to zero for negative *F*^2^. The threshold expression of *F*^2^ > σ(*F*^2^) is used only for calculating *R*-factors(gt) *etc*. and is not relevant to the choice of reflections for refinement. *R*-factors based on *F*^2^ are statistically about twice as large as those based on *F*, and *R*- factors based on ALL data will be even larger.

### Fractional atomic coordinates and isotropic or equivalent isotropic displacement parameters (Å^2^)

**Table:**

	*x*	*y*	*z*	*U*_iso_*/*U*_eq_	
C1	1.0028 (7)	0.8829 (5)	−0.2061 (3)	0.0777 (15)	
H1	1.0615	0.8029	−0.2241	0.093*	
C2	0.8502 (7)	1.0221 (5)	−0.1387 (3)	0.0888 (17)	
H2	0.7824	1.0569	−0.1010	0.107*	
C3	0.9580 (6)	0.6644 (4)	−0.0936 (2)	0.0632 (12)	
H3	1.0140	0.6437	−0.1368	0.076*	
C4	0.9482 (6)	0.5533 (4)	−0.0388 (2)	0.0562 (11)	
C5	0.8680 (7)	0.5730 (4)	0.0283 (2)	0.0667 (13)	
H5	0.8150	0.6611	0.0384	0.080*	
C6	0.8654 (6)	0.4652 (4)	0.0798 (2)	0.0629 (13)	
H6	0.8085	0.4815	0.1234	0.076*	
C7	0.9468 (6)	0.3314 (4)	0.0680 (2)	0.0582 (11)	
C8	1.0235 (6)	0.3114 (4)	−0.0006 (2)	0.0635 (12)	
H8	1.0746	0.2231	−0.0117	0.076*	
C9	1.0236 (6)	0.4195 (4)	−0.0505 (2)	0.0642 (12)	
H9	1.0770	0.4026	−0.0948	0.077*	
C10	1.0615 (7)	0.0993 (4)	0.1119 (2)	0.0684 (13)	
H10A	1.1077	0.0746	0.1591	0.082*	
H10B	1.1583	0.1240	0.0810	0.082*	
C11	0.9734 (7)	−0.0308 (4)	0.0814 (3)	0.0824 (15)	
H11A	0.8749	−0.0546	0.1106	0.124*	
H11B	1.0523	−0.1110	0.0809	0.124*	
H11C	0.9362	−0.0106	0.0329	0.124*	
C12	0.8512 (7)	0.2348 (5)	0.1858 (2)	0.0754 (14)	
H12A	0.8145	0.1383	0.1997	0.091*	
H12B	0.7483	0.2917	0.1765	0.091*	
C13	0.9488 (9)	0.3017 (6)	0.2477 (3)	0.112 (2)	
H13A	1.0464	0.2421	0.2596	0.168*	
H13B	0.8742	0.3094	0.2890	0.168*	
H13C	0.9882	0.3964	0.2341	0.168*	
N1	0.8934 (7)	1.0979 (5)	−0.1959 (3)	0.1007 (15)	
N2	0.9902 (7)	1.0065 (5)	−0.2379 (2)	0.0907 (14)	
N3	0.9164 (5)	0.8878 (4)	−0.1417 (2)	0.0651 (10)	
N4	0.8933 (5)	0.7886 (4)	−0.08490 (19)	0.0679 (11)	
N5	0.9521 (5)	0.2252 (3)	0.11934 (19)	0.0654 (10)	

### Atomic displacement parameters (Å^2^)

**Table:**

	*U*^11^	*U*^22^	*U*^33^	*U*^12^	*U*^13^	*U*^23^
C1	0.109 (5)	0.067 (3)	0.057 (3)	−0.009 (3)	−0.003 (3)	−0.004 (2)
C2	0.103 (5)	0.079 (3)	0.084 (4)	0.022 (3)	0.012 (3)	0.010 (3)
C3	0.077 (3)	0.069 (3)	0.044 (3)	−0.002 (3)	0.002 (2)	−0.001 (2)
C4	0.065 (3)	0.057 (2)	0.047 (2)	−0.003 (2)	0.001 (2)	−0.0034 (19)
C5	0.085 (4)	0.056 (2)	0.059 (3)	0.005 (2)	0.006 (2)	−0.008 (2)
C6	0.081 (4)	0.062 (3)	0.045 (3)	0.005 (2)	0.012 (2)	−0.004 (2)
C7	0.064 (3)	0.061 (2)	0.049 (3)	−0.006 (2)	0.002 (2)	−0.005 (2)
C8	0.075 (3)	0.060 (2)	0.056 (3)	0.007 (2)	0.006 (2)	−0.004 (2)
C9	0.068 (3)	0.069 (3)	0.055 (3)	0.001 (2)	0.010 (2)	−0.003 (2)
C10	0.079 (3)	0.069 (3)	0.057 (3)	0.010 (3)	0.001 (2)	0.010 (2)
C11	0.097 (4)	0.073 (3)	0.077 (4)	0.008 (3)	−0.006 (3)	−0.003 (3)
C12	0.092 (4)	0.077 (3)	0.058 (3)	−0.008 (3)	0.011 (3)	−0.001 (2)
C13	0.143 (6)	0.140 (4)	0.053 (3)	−0.027 (5)	0.005 (4)	−0.024 (3)
N1	0.117 (4)	0.082 (3)	0.104 (4)	0.009 (3)	0.003 (3)	0.025 (3)
N2	0.125 (4)	0.078 (3)	0.070 (3)	−0.016 (3)	−0.002 (3)	0.012 (2)
N3	0.081 (3)	0.061 (2)	0.054 (2)	−0.002 (2)	0.000 (2)	0.0043 (18)
N4	0.087 (3)	0.061 (2)	0.056 (2)	0.002 (2)	0.002 (2)	0.0061 (19)
N5	0.081 (3)	0.067 (2)	0.049 (2)	0.005 (2)	0.009 (2)	0.0040 (18)

### Geometric parameters (Å, °)

**Table:**

C1—N2	1.288 (6)	C8—H8	0.9300
C1—N3	1.366 (6)	C9—H9	0.9300
C1—H1	0.9300	C10—N5	1.445 (5)
C2—N1	1.313 (6)	C10—C11	1.493 (6)
C2—N3	1.344 (5)	C10—H10A	0.9700
C2—H2	0.9300	C10—H10B	0.9700
C3—N4	1.262 (5)	C11—H11A	0.9600
C3—C4	1.444 (5)	C11—H11B	0.9600
C3—H3	0.9300	C11—H11C	0.9600
C4—C9	1.384 (6)	C12—N5	1.459 (6)
C4—C5	1.400 (6)	C12—C13	1.505 (7)
C5—C6	1.378 (6)	C12—H12A	0.9700
C5—H5	0.9300	C12—H12B	0.9700
C6—C7	1.405 (5)	C13—H13A	0.9600
C6—H6	0.9300	C13—H13B	0.9600
C7—N5	1.366 (5)	C13—H13C	0.9600
C7—C8	1.412 (6)	N1—N2	1.369 (6)
C8—C9	1.361 (5)	N3—N4	1.406 (4)
			
N2—C1—N3	109.4 (5)	N5—C10—H10B	108.6
N2—C1—H1	125.3	C11—C10—H10B	108.6
N3—C1—H1	125.3	H10A—C10—H10B	107.6
N1—C2—N3	111.2 (5)	C10—C11—H11A	109.5
N1—C2—H2	124.4	C10—C11—H11B	109.5
N3—C2—H2	124.4	H11A—C11—H11B	109.5
N4—C3—C4	122.4 (4)	C10—C11—H11C	109.5
N4—C3—H3	118.8	H11A—C11—H11C	109.5
C4—C3—H3	118.8	H11B—C11—H11C	109.5
C9—C4—C5	116.2 (4)	N5—C12—C13	113.4 (4)
C9—C4—C3	120.2 (4)	N5—C12—H12A	108.9
C5—C4—C3	123.6 (4)	C13—C12—H12A	108.9
C6—C5—C4	121.7 (4)	N5—C12—H12B	108.9
C6—C5—H5	119.1	C13—C12—H12B	108.9
C4—C5—H5	119.1	H12A—C12—H12B	107.7
C5—C6—C7	121.4 (4)	C12—C13—H13A	109.5
C5—C6—H6	119.3	C12—C13—H13B	109.5
C7—C6—H6	119.3	H13A—C13—H13B	109.5
N5—C7—C6	122.5 (4)	C12—C13—H13C	109.5
N5—C7—C8	121.2 (4)	H13A—C13—H13C	109.5
C6—C7—C8	116.3 (4)	H13B—C13—H13C	109.5
C9—C8—C7	121.0 (4)	C2—N1—N2	105.5 (4)
C9—C8—H8	119.5	C1—N2—N1	109.2 (5)
C7—C8—H8	119.5	C2—N3—C1	104.7 (4)
C8—C9—C4	123.3 (4)	C2—N3—N4	121.5 (4)
C8—C9—H9	118.4	C1—N3—N4	133.8 (4)
C4—C9—H9	118.4	C3—N4—N3	116.6 (4)
N5—C10—C11	114.6 (4)	C7—N5—C10	121.9 (3)
N5—C10—H10A	108.6	C7—N5—C12	121.8 (4)
C11—C10—H10A	108.6	C10—N5—C12	116.3 (3)
			
N4—C3—C4—C9	−179.0 (5)	N1—C2—N3—C1	−1.1 (6)
N4—C3—C4—C5	−0.4 (7)	N1—C2—N3—N4	177.3 (4)
C9—C4—C5—C6	0.4 (7)	N2—C1—N3—C2	1.0 (6)
C3—C4—C5—C6	−178.3 (4)	N2—C1—N3—N4	−177.2 (4)
C4—C5—C6—C7	1.5 (7)	C4—C3—N4—N3	178.2 (4)
C5—C6—C7—N5	177.2 (4)	C2—N3—N4—C3	178.8 (5)
C5—C6—C7—C8	−3.0 (7)	C1—N3—N4—C3	−3.3 (7)
N5—C7—C8—C9	−177.5 (4)	C6—C7—N5—C10	−168.6 (4)
C6—C7—C8—C9	2.7 (7)	C8—C7—N5—C10	11.6 (6)
C7—C8—C9—C4	−1.0 (7)	C6—C7—N5—C12	9.5 (6)
C5—C4—C9—C8	−0.6 (7)	C8—C7—N5—C12	−170.2 (4)
C3—C4—C9—C8	178.1 (4)	C11—C10—N5—C7	−96.1 (5)
N3—C2—N1—N2	0.8 (7)	C11—C10—N5—C12	85.7 (5)
N3—C1—N2—N1	−0.5 (6)	C13—C12—N5—C7	−92.9 (5)
C2—N1—N2—C1	−0.2 (7)	C13—C12—N5—C10	85.3 (5)

### Hydrogen-bond geometry (Å, °)

**Table:**

*D*—H···*A*	*D*—H	H···*A*	*D*···*A*	*D*—H···*A*
C1—H1···N1^i^	0.93	2.43	3.296 (7)	155

Symmetry codes: (i) −*x*+2, *y*−1/2, −*z*−1/2.
